# A large-scale benchmark study of tools for the classification of protein-coding and non-coding RNAs

**DOI:** 10.1093/nar/gkac1092

**Published:** 2022-11-24

**Authors:** Dalwinder Singh, Joy Roy

**Affiliations:** National Agri-Food Biotechnology Institute, SAS Nagar, Punjab, 140306, India; National Agri-Food Biotechnology Institute, SAS Nagar, Punjab, 140306, India

## Abstract

Identification of protein-coding and non-coding transcripts is paramount for understanding their biological roles. Computational approaches have been addressing this task for over a decade; however, generalized and high-performance models are still unreliable. This benchmark study assessed the performance of 24 tools producing >55 models on the datasets covering a wide range of species. We have collected 135 small and large transcriptomic datasets from existing studies for comparison and identified the potential bottlenecks hampering the performance of current tools. The key insights of this study include lack of standardized training sets, reliance on homogeneous training data, gradual changes in annotated data, lack of augmentation with homology searches, the presence of false positives and negatives in datasets and the lower performance of end-to-end deep learning models. We also derived a new dataset, RNAChallenge, from the benchmark considering hard instances that may include potential false alarms. The best and least well performing models under- and overfit the dataset, respectively, thereby serving a dual purpose. For computational approaches, it will be valuable to develop accurate and unbiased models. The identification of false alarms will be of interest for genome annotators, and experimental study of hard RNAs will help to untangle the complexity of the RNA world.

## INTRODUCTION

The classification of coding and non-coding RNAs (ncRNAs) is a complex task due to their overlapping characteristics and functions. Since the unravelling of the RNA world began, this problem not only baffled biologists but also posed a significant challenge for machine learning (ML) approaches. In the initial decades, coding RNAs remained in the spotlight as they serve as a template to create proteins for carrying out vital cellular functions. The residual RNA elements, termed ncRNAs, received little attention and were usually considered noise. However, a fundamental shift occurred in this domain with the discovery of ncRNAs with relevant functions. A small share of the coding regions, for instance ∼2% in the mammalian genome, motivated many researchers to look into vast alternative regions to understand genomic evolution (1), to inspect the role of cellular functions (1,2), to find a cure for various diseases (3,4) and to search for unanswered biological questions and mechanisms (5,6). Subsequently, ncRNAs were identified with roles beyond translation and ribosomal activities. Now, ncRNAs can be categorized into microRNAs (miRNAs), small nuclear RNAs (snRNAs), small nucleolar RNAs (snoRNAs), Piwi-interacting RNAs (piRNAs), small Cajal body-associated RNAs (scaRNAs), long intergenic non-coding RNAs (lincRNAs), antisense, sense overlapping, sense intronic, and so on. These classes have significant variations in length, functions, occurrence, structures and distributions across species (7).

The discovery of millions of transcripts using next-generation sequencing technologies from different life forms makes their characterization paramount to thoroughly understanding the working mechanisms of living cells. This tremendous increase in the volume of transcriptome data turned researchers towards computational approaches, which are effective, flexible, scalable and efficient for predicting the coding potential. These approaches rely on the different traits of transcripts and ML algorithms to discover the patterns for distinguishing the coding transcripts from other types. However, this binary classification problem has proved to be more challenging than anticipated as experimentally validated RNAs are displacing old classification rules (8) that were supposed to generalize the task. Several common characteristics have been found between two classes, including open reading frames (ORFs), similar structure and overlapping of functions and genomic locations (9). Additionally, the problem domain is gradually transforming with the availability of more high-quality data. The key drivers responsible for changes are evolutionary pressures, recovery of partial length transcripts, better detection of small ORFs and correction of annotation errors.

Over a decade of research, many classification tools have been developed by fusing RNA descriptors and experimenting with ML algorithms. The descriptors were developed from the nucleotide and amino acid sequences (10–13), similarity searches on protein databases (14–16) and reference genomes (17–20). Sequence-based descriptors are the most popular approach for building the models due to their low computational cost, good performance and generalization to different life forms. These descriptors capture the intrinsic information of sequences without relying on the comprehensive annotation of reference genomes or their alignments with massive databases to solve this task. Thus, sequence-based tools are the primary focus of this study.

The previous benchmarking efforts to investigate this problem are limited to a few models and datasets. Such efforts include the comparison of pre-trained *Homo sapiens* models of LncRNAnet (21), LncADeep (16) and LncFinder (22) tools on full human and mouse transcriptomes (23). Choosing *H. sapiens* for comparison is a poor choice, however, because models were also trained with different subsets of the same species. It hides the true potential of tools as highly overlapping training and test sets can yield higher performance, and vice versa. The reported performances of LncADeep, LncRNAnet and LncFinder tools, in descending order, correlated with >50 000, 42 000 and 16 000 transcripts used for training models in humans. In contrast, LncFinder performed better than LncRNAnet on mouse species (24). In another study, Duan *et al.* (25) compared 41 models from 14 tools on three *H. sapiens*, two *Mus musculus*, one *Oncorhynchus mykiss* and one *Hippocampus comes* dataset sampled randomly with 10 000 transcripts, and one multispecies dataset constructed from 33 species by selecting up to 2000 RNAs from each species. Their study raised the issue of overlapped training and test sets, performed experiments with erroneous transcripts and analysed performance with the joint prediction of models and recommended models depending upon situations and taxonomic levels. However, small test sets cannot ensure the robustness of situational models. Zheng *et al.* (26) compared the performance of 17 tools on two relatively large *H. sapiens* datasets and one dataset constructed from 30 species with criteria similar to (25). However, this benchmark suffers from the overlapping problem because models were trained with *H. sapiens* and also lacks the latest tools. Xu *et al.* (27) combined nine methods in a single package, ezLncPred, for the classification of RNAs, but did not compare them. Klapproth *et al.* (28) performed a mini-benchmark with five tools and one dataset comprising 400 RNA sequences. Thus, the existing studies are insufficient to pinpoint the key factors impeding the performances of various tools even though the absence of superior tools for predicting coding potential was suggested.

This study presents a systematic benchmark using 24 popular RNA classification tools on 135 small and large transcriptomic datasets from existing studies. These datasets cover a wide range of species (49) and have several heterogeneous datasets. For the comprehensive analysis of tools, experiments are performed with 58 models trained from homogeneous and heterogeneous data sources. The potential bottlenecks hindering the performance of these tools are identified. Specifically, we focused on the absence of a gold standard training set due to gradual changes in the transcriptome data. It made *H. sapiens* a *de facto* source for building, evaluating and comparing the tools, but produces biased outcomes due to overlapped training and test sets. This approach also complicates the analysis when using species-specific models for comparison. In addition, the essence of training sources, i.e. benefits of heterogeneous data collected from various species over homogeneous data taken from single species, for constructing models requires a close examination since ML and deep learning (DL) algorithms are sensitive to the input data. The classifiers may not fit well on poorly chosen data supporting their careful selection for this problem. Another important concern is the augmentation of sequence-intrinsic models with similarities found in high-quality databases. A few tools have opted for this approach and reported better performance on several species if not all. Lastly, identifying hard RNAs, i.e. consistently misclassified instances, is vital to assess the limitations of current tools and pave the way for developing the next-generation classification methods. For this purpose, we built a new validation set, RNAChallenge, from the benchmark and assessed the performance of all models separately. This validation set is also helpful in detecting false positives and false negatives, a problem commonly found in the biological domain. Overall, we showed significant room for improving the tools by addressing these issues.

## MATERIALS AND METHODS

### Materials

**Figure 1.**
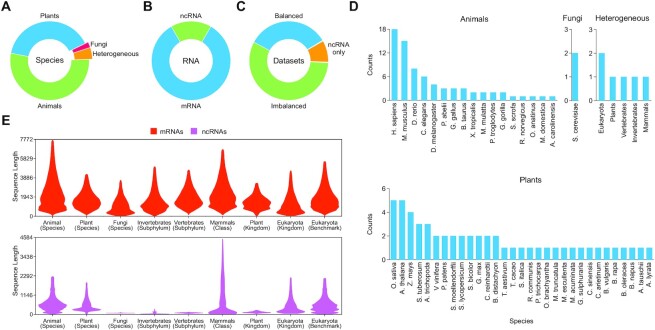
Information about the datasets used in the benchmarking. (**A**) The kingdom-wise coverage of species and heterogeneous datasets, (**B**) share of mRNAs and ncRNAs, and (**C**) balanced and imbalanced datasets. (**D**) Taxonomic distribution of species at the kingdom level and heterogeneous datasets at their primary levels. (**E**) Length distribution of sequences at species, subphylum, class, kingdom and domain levels.

We have collected 135 test datasets from the spectrum of species to ensure the wide-range applicability of the present study. These datasets belong to animal, plant and fungal kingdoms and are acquired from existing methods in order to maintain the data quality during experiments. The methods comprise CPAT (1) (10), PLEK (9) (29), PLncPRO (16) (15), CPC2 (6) (30), FEELnc (12) (31), mRNN (5) (11), LncRNAnet (2), LncADeep (2), LncFinder (6), CPPred (12) (32), CNIT (35) (33), RNAplonc (8) (34), LGC (10) (35), PreLnc (6) (36), RNAC (4) (20) and DeepCPP (1) (13). The datasets are pre-processed by removing characters other than the ‘ACGT’ (23). Figure 1 provides a comprehensive analysis of datasets to highlight the current state of the computational approaches to solve this problem. Although RNAs are ubiquitous, tools which have been developed and evaluated centre around animal and plant kingdoms as shown in the kingdom-wise coverage of species (Figure 1A). The total share of mRNAs and ncRNAs, as shown in Figure 1B, also reveals the under-representation of non-coding transcripts and skewness towards the coding elements owing to tremendous efforts in their search. Presently, mRNAs act as the prime contributor in training and validation sets, but their significant share undermines the theoretical foundations of RNA that hold the non-coding elements as the majority class. Consequently, validation datasets are also imbalanced as shown in Figure 1C. The benchmark comprises 46 balanced, 77 imbalanced and 12 ncRNA-only datasets. Figure 1D shows the distribution of species where *H. sapiens* and *M. musculus* in the animal kingdom, and *Oryza sativa* and *Arabidopsis thaliana* in plants outweigh other species in experiments. Refer to Supplementary File S1 for detailed information on the datasets. Length distribution of transcripts after removing the outliers, Figure 1D shows the disparity among mRNA and ncRNA classes at species, subphylum, class, kingdom and domain levels. Refer to Supplementary Figure S1 for full-length plots. The longer mRNAs but shorter ncRNAs indicate the possibility of a far greater number of non-coding transcripts within the same genomic space. It also implies a problem of greater complexity of RNA classification than expected since the share of ncRNA exceeds 90% in most species.

### Methods

**Figure 2.**
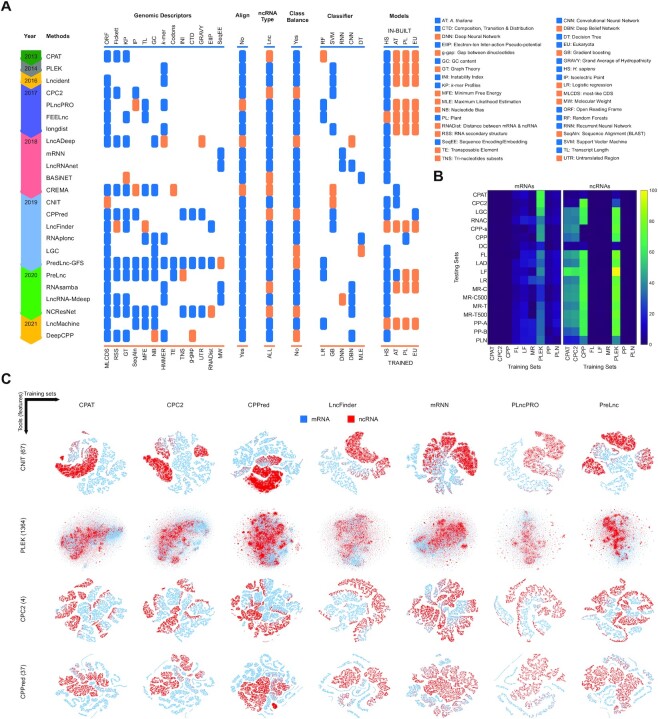
A summary of 24 tools for the classification of mRNAs and ncRNAs. (**A**) The timeline of tools is provided along with genomic descriptors for feature extraction, sequence alignments with databases, type of non-coding RNAs, class imbalance problem, classifiers and training sources to test the benchmark. (**B**) Similarity of nine *H. sapiens* training sets with 18 benchmark sets. (**C**) t-SNE plots showing the classification capabilities of four tools on seven human training sets.

We have considered 24 classification tools available in the public domain for empirical analysis. These methods are CPAT, PLEK, CPC2, Lncident (LI) (37), PLncPRO (PP), FEELnc (FL), longdist (LD) (38), LncADeep (LAD), mRNN (MR), LncRNAnet (LR), BASiNET (BI) (39), CREMA (CRE) (40), CNIT, CPPred (CPP), LncFinder (LF), RNAplonc (RP), LGC, PredLnc-GFStack (PGF) (41), PreLnc (PLN), RNAsamba (RS) (12), LncRNA-Mdeep (LMD) (42), NCResNet (NCR) (43), LncMachine (LM) (44) and DeepCPP (DC). A brief discussion of the methods is provided in Supplementary Section 2. The key characteristics of these methods are summarized in Figure 2A. Overall, most tools share the genomic descriptors to differentiate mRNAs and ncRNAs substantially. These methods rely on the fusion of nucleotide descriptors such as the ORF, Fickett score, *k*-mer profiles of mRNAs and ncRNAs (especially hexamer score), transcript length, GC content, *k*-mer and its variants such as *g*-gap, codons, composition, transition and distribution (CTD), nucleotide bias, transposable elements, tri-nucleotide subsets, untranslated regions and distance between mRNAs and ncRNAs. The protein descriptors include isoelectric point, molecular weight, instability index and grand average of hydropathicity (GRAVY). The physicochemical descriptors of RNA derived with electron–ion interaction pseudo-potential (EIIP) and RNA secondary structure descriptors are also used in few tools for feature extraction. The nucleotide-based most-like coding sequence (CDS) descriptor has been utilized in CNIT, whereas BI used graph theory to extract topological features from complex networks. The sequence encoding and embedding techniques have been employed in mRNN and LR tools to extract the features automatically with DL. PP, LAD and CRE tools also rely on protein databases to find similarities with known proteins. Undoubtedly, these methods have the edge over the remaining approaches as conserved and known sequences can be directly corroborated from databases. Nevertheless, differences in performance are probably due to dissimilar classification boundaries arising from the quality and quantity of training data as well as the classifier used for building models. We have used DIAMOND (45) to perform sequence searches in the UNIPROT database (46) for PP and CRE tools, whereas HMMER is used in LAD to search sequences against the Pfam 29 database (47).

The type of non-coding transcripts, i.e. lncRNAs with lengths >200 nt or ncRNAs without length bounds, used in training indicates the coverage of non-coding classes. The lncRNAs usually consist of rRNAs, lincRNAs, antisense, sense intronic and sense overlapping transcripts, while ncRNAs represent all known transcripts without coding potential. It also includes miRNAs, piRNAs, tRNAs, snRNAs, snoRNAs and other small ncRNAs. The imbalanced training data highlight the susceptibility of tools to be biased towards the majority class. The classifier used to train the models and source of training data show the diversity of approaches to solving this problem. We have used existing models and constructed several new ones for this exhaustive analysis. Specifically, we selected the training sets from four sources to standardize the learning process. Two species-, one kingdom- and one domain-level dataset are supplied to build models wherever the training mechanism of tools is available but the prediction model was not built. These comprise species-level *H. sapiens* (HS) and *A. thaliana* (AT) datasets from CNIT, a kingdom-level plant (PL) dataset from RP and a domain-level dataset from CPP tools. The PL dataset was constructed from *A. thaliana*, *Crocus sativus*, *Glycine max*, *Populus trichocarpa* and *O. sativa*, whereas the Eukaryota (EU) dataset was formed from *H. sapiens*, *M. musculus*, *Danio rerio*, *Drosophila melanogaster*, *Saccharomyces cerevisiae*, *Caenorhabditis elegans* and *A. thaliana*. All methods have a human model except for CRE and RP which were developed for plant species and lack training pipelines. Further, LM has been retrained using the CNIT *H. sapiens* dataset in the absence of training datasets and models. Both FL and LF have been retrained with their corresponding datasets. Overall, we obtained 58 models from all tools, including two models tackling partial transcripts, for empirical assessment. We used the holdout approach at the tools level to carry out this benchmark in which 18 tools (75%) yielding 49 models have been used for comparative analysis to find factors impeding performance and to construct a new dataset. In the second set of experiments, six tools producing nine models validate the selection of hard instances in the challenging dataset.

Figure 2B shows the overlapping of training sets of *H. sapiens* species with the test sets in the benchmark. We used the CD-HIT tool (48) with 0.9 similarity threshold to analyse the redundancy between nine training and 18 test sets. CPAT, CPC2 and PLEK tools in ncRNAs and FL, LF and MR tools in mRNAs have a higher overlap with test sets. Figure 2C illustrates the t-SNE plots of handcrafted features from CNIT (67), PLEK (1364), CPC2 (4) and CPP (37) tools that are extracted from seven different sources of human training sets. Each two-dimensional coordinate indicates a projected map of an instance with *n* features. The variations in the overlapping of classes in these plots highlight the discrepancies in classification boundaries from different training sets. It also provides general insights into the performance of tools by showing the discrimination capabilities of descriptors through class overlapping. For instance, significant overlapping exhibited by PLEK is likely to yield a lower performance than the other three tools on the benchmark.

## RESULTS

We have considered several factors for in-depth analysis of this classification problem. They include performance analysis based on organism taxonomy, the impact of training sources on the performance of tools, analysis of hard examples from the whole benchmark and superiority of learning mechanisms in developing the state-of-the-art models. Initially, models are compared based on taxonomic categories as shown in Figure 1D. At the species level, HS and AT are considered, whereas the remaining species belonging to animals, plants and fungi are analysed at the kingdom level. The heterogeneous datasets constructed at different taxonomic levels have been considered separately. The independent assessment revealed the lack of superior tools, complications with the evaluation of HS data and incapabilities of models to retain high performance on a large scale. Secondly, we showed that heterogeneous training data are superior to homogeneous data by investigating the impact of standard training sources on the discrimination capabilities of tools. Thirdly, visual investigations of classification decisions obtained from these tools highlighted the possibility of false alarms in the benchmark. It also helps to construct the RNAChallenge dataset comprising mRNAs and ncRNAs with low confidence scores. Lastly, end-to-end DL approaches have been investigated in contrast to traditional learning mechanisms owing to their unchallenged performance in the artificial intelligence (AI) domain. Currently, these approaches fail to beat the conventional combination of handcrafted features and ML algorithms for solving this problem.

Several metrics are used to compare models on the benchmark by considering ncRNAs as a positive class and mRNAs as a negative class. Accuracy, precision, recall, F1-score (FSC) and Matthews Correlation Coefficient (MCC) rates are calculated with the correctly classified ncRNAs (TP), correctly classified mRNAs (TN), incorrectly classified ncRNAs (FN) and incorrectly classified mRNAs (FP). It is given as follows:

$$\begin{eqnarray*} Accuracy = \frac{TP+TN}{TP+FP+FN+TN} \end{eqnarray*}$$

$$\begin{eqnarray*} Precision = \frac{TP}{TP+FP} \end{eqnarray*}$$

$$\begin{eqnarray*} Recall = \frac{TP}{TP+FN} \end{eqnarray*}$$

$$\begin{eqnarray*} F1-score = \frac{2\times TP}{2\times TP + FP + FN} \end{eqnarray*}$$

$$\begin{eqnarray*} MCC = \frac{TP\times TN - FP \times FN}{\sqrt{(TP+FP)(TP+FN)(TN+FP)(TN+FN)}} \end{eqnarray*}$$

The models are compared with the FSC because accuracy is a poor choice due to the majority of imbalanced datasets, area under curve (AUC) scores are not available for all tools and MCC is not helpful when dealing with one-class ncRNAs datasets.

### Statistical analysis

While comparing the multiple RNA classification tools over many datasets, the statistical analysis of their performance outcomes becomes essential. Several parametric and non-parametric statistical tests are available in the literature, but the Friedman test (49) has become a standard for analysing multiple classifiers over several datasets (50). It is a non-parametric approach to compare the methods by ranking them for each dataset separately using a performance measure. The test evaluates the null hypothesis that the ranks of different models are equal when evaluated on the same datasets. Here, it is used to compare the models on the validation datasets where the best performing method has the lowest rank, and so forth. However, this test fails to identify which methods are significantly different from each other. So, an additional post-hoc test is required to determine the differences from the baseline method (i.e. best performing method). For this purpose, the Holm post-hoc test has been utilized which compares the baseline with the remaining methods. The test takes the ordered Friedman ranks (*R*_1_ ≤ *R*_2_ ≤ ... ≤ *R*_*N*_) and computes $z = R_i - R_1/\sqrt{N(N+1)/6M}$ to obtain sorted *P*-values (*P*_1_, *P*_2_,..., *P*_*N*_), where *R*_1_ denotes the control model, *N* denotes total models and *M* denotes the datasets. Then, a step-down procedure is used to reject or accept the null hypothesis by comparing *P*_*i*_ with α/(*N* − *i*). Firstly, *P*_1_ is compared with α/(*N* − 1) and the hypothesis is rejected if it is less. Next, *P*_2_ is tried and the hypothesis is rejected if <α/(*N* − 2). The process is repeated for higher values. Once a hypothesis is accepted, the following hypotheses are also retained. This test is not used while analysing the outcomes at taxonomic levels as models exceed the datasets. Instead, post-hoc analysis is performed on the whole benchmark only.

### Benchmarking models

**Figure 3.**
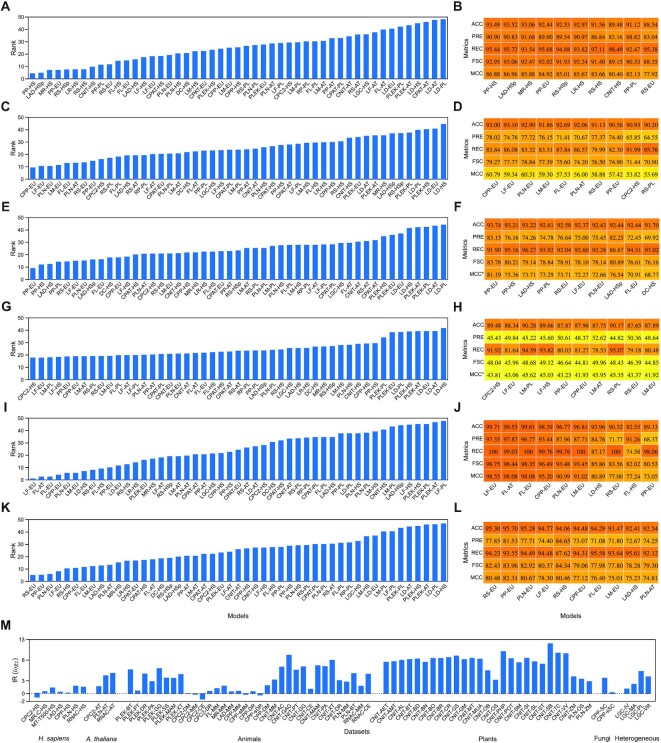
Comparative analysis of models using the FSC and prediction outcomes of top performing models. (**A**) Friedman ranks and (**B**) top models on *H. sapiens* datasets. (**C**) Friedman ranks and (**D**) top models on *A. thaliana* datasets. (**E**) Friedman ranks and (**F**) top models on animal datasets except *H. sapiens*. (**G**) Friedman ranks and (**H**) top models on plant datasets except *A. thaliana*. (**I**) Friedman ranks and (**J**) top models on fungi datasets. (**K**) Friedman ranks and (**L**) top models on integrated datasets. (**M**) Imbalance ratio of datasets having unequal mRNAs and ncRNAs.

Figure 3 presents the outcomes of the Friedman test, top performing models and the imbalance ratio (IR) of datasets for different scenarios. The findings of the comparative analysis are presented on species, kingdom and integrated datasets to show the shortcomings of current methods to characterize the RNAs.

### Benchmarking models on species-level datasets (*H. sapiens* and *A. thaliana*)

Initially, HS (Supplementary File S2) and AT (Supplementary File S3) datasets are used for comparison owing to their widespread use in building models. Figure 3A presents the Friedman ranks of all models for HS. The *P*-value of 3 × 10^−104^ (<0.05 assumed significance level) shows that the performances of models are independent and have significant differences in outcomes. Overall, the ranks on 18 datasets indicate the superiority of alignment-augmented models, failure of plant-based models and bias in outcomes due to overlapping training and test sets.

The similarity searches on UniProt and Pfam databases boosted the performances of PP and LAD methods by validating many mRNAs directly (Figure 3B). As a result, these hybrid tools excelled in recognizing RNAs as compared with others. The LAD and RS models for partial-length transcripts also attained high performance. It necessitates the integration of such traits into the tools to deal with issues associated with the short read RNA-sequencing technologies. In addition, the models built with the same species captured the characteristics of RNAs much better than other species and integrated datasets. When analysing the general trend for training sources on model performance, substantial differences in performances of HS- and EU-based models are observed in contrast to AT- and PL-based models. These results indicate the high sensitivity of models towards the same species. The impact of the class imbalance problem is minor, as only seven out of 18 datasets suffer from this problem (Figure 3M) which agrees with the high precision and recall rates (>80% and 90%) of the top-performing models. However, tools (PLEK, LAD, RS and LR) trained with randomly selected large datasets became more sensitive to human species because of similarities in training and test sets. This issue has been elaborated further.

Figure 3C provides the ranks for AT datasets where the obtained *P*-value (5 × 10^−5^) indicates the significant difference in performances of models. These ranks reveal the high performance of models trained with integrated training datasets, whereas the small sized AT training set and alignment-augmented tools yield suboptimal performance. With the newly constructed models from the EU training set, several tools (CPP, LF, PLN, LM, LF, RS and PP) obtained higher performance than the pre-trained models available (Figure 3D). Similarly, several tools gained higher performance with PL-based training sets than their corresponding AT and HS sources. These findings indicate that heterogeneous training sources are more appropriate for building models than homogeneous sources. Interestingly, our balanced training set yields a lower performance than the pre-trained PLN-AT model and it also shows the sensitivity of training data on classification decisions. The top models trained with the EU dataset correctly classified the majority class (AVV >90%), but had a low recall (≤88%), precision (70–78%), FSC (≤80%) and MCC (≤60%) rates. Alternatively, CPC2-HS and RS-PL models favour ncRNAs with a high recall rate (>90%) but have low precision (<66%). These models are susceptible to bias in training data and are inclined towards either class due to high IR of CPC2, PLN and RNAC datasets, as shown in Figure 3M. Moreover, the alignment-augmented PP-PL model is unable to correctly identify transcripts, and best performing models on HS, including partial ones, fail to achieve top ranks. This validates the difficulties in retaining the performances of tools cross-kingdoms. Furthermore, the majority of ncRNAs in the RNAC dataset are detected correctly by >50% of models. However, every model has misclassified at least 10% transcripts of the FL ncRNA-only dataset.

### Benchmarking models on kingdom level datasets

In the second set of experiments, models are compared on datasets belonging to animal, plant and fungi kingdoms except for HS and AT species. The detailed outcomes of models are provided in Supplementary Files S4 for animals, S5 for plants and S6 for fungi. The Friedman ranks on the animal benchmark are presented in Figure 3E, and the obtained *P*-value of 1 × 10^−172^ shows the significant differences in performances. Overall, the ranks highlight better performance with alignment-augmented methods and integrated training sets, and inadequate discrimination capabilities of PL models and biased models. The heterogeneous training sources emerged as a superior source for developing models on most tools. For instance, EU-based models of RS, LF, PLN and FL tools made it into the top 10 while their second-best models attained ranks of 16, 12, 14 and 21. Also, only three tools (PP, LAD and RS) are common with top-performing tools on HS datasets, implying the high specificity of MR, LR and CNIT tools for human species. These tools also lack models trained with heterogeneous data and so do not generalize well with only the human transcriptome. Undoubtedly, reliance on organism-level training sources limits the potential of tools considerably as cross-species diversity of transcripts cannot be tackled effectively. The poor performance with AT- and most PL-based models is another indicator of performance bottlenecks due to RNA diversity at kingdom levels. The widely used Uniprot and Pfam databases host proteins from animal species mainly, and thereby help to recognize mRNAs more precisely (Figure 3F). Searching for sequence homology benefited three out of four PP and both LAD models, which bolstered their role as a critical genomic descriptor to recognize RNA correctly. Moreover, these findings match with the trend observed in HS datasets. The unusually high IR (maximum value is 997) of these datasets, as shown in Figure 3M, reflects the current knowledgebase where mRNAs vastly exceed the ncRNAs in several species. In the presence of such imbalanced datasets, the PP-EU model demonstrates balanced classification of RNAs as compared with its following six models (Figure 3F), with significant margins in precision (>6%), FSC (>3%) and MCC (>5%) rates. The coding transcripts of *C. elegans* from the CNIT and CPC2 tools have been identified correctly with FL-AT, FL-EU, CPP-EU and LF-EU models. On the other hand, non-coding sets of LF-DR, RNAC-CE, LGC-CE, all sets of PLEK except MM and DR, and all animal sets of CNIT except MM and DR have been identified correctly with at least one model. The high performance of models on these datasets is primarily attributed to their small size as there are close to 3500 mRNAs in *C. elegans* only whereas several ncRNAs datasets have <500 instances. In the case of FL ncRNA-only sets, the best models correctly recognized >90% of instances except for CE and DM species.

The ranks of models on datasets from the plant kingdom are shown in Figure 3G, and the obtained *P*-value of their comparison is also significant (4 × 10^−85^). Overall, the outcomes reveal poor performance by all models in recognizing ncRNA, lower performance with alignment-augmented tools and higher performance with integrated training sources. Firstly, ranks of best models are too high (close to 20), indicating their failure to retain the performance across all datasets. The precision, FSC and MCC rates of models less than 53%, 50% and 46% indicate the considerable misclassification of mRNAs as ncRNAs. That is why, irrespective of poor performance, the accuracy of top models exceeds 87% as shown in Figure 3H. Further inspection of top-ranked models shows the correlation between biased models and training sources. Some models are biased toward mRNAs (LF-EU, PP-EU, CPP-EU, LM-AT, RS-EU and LM-EU) while others favoured ncRNAs (CPC2-HS, LNM-PL, LF-HS and RS-PL). The EU- and AT-based models favoured the recognition of mRNA, whereas HS- and PL-based sources detected more ncRNAs. When comparing all four training sources, models developed with integrated datasets outperformed the species-level datasets. Secondly, the plant datasets have a huge class disparity similar to those of animals. In the 27 class-imbalanced plant datasets (Figure 3M), IR is >20 and reaches as high as 7800 (CNIT-SB). Thus, most pre-trained and newly constructed models do not fit well on the validation datasets. Aligning sequences against popular databases is ineffective for plants as only one model made it into the top 10. In the absence of similar homologous transcripts in the sequence databases, the performance of augmented models is low. This finding is further validated by comparing the best tools at AT and PL levels. Several tools, namely CPC2, CPP, LF, LM, PP and RS, attained similar performances. So, repetition of models at a large scale, even though moderately accurate, implies more classification complexity for plant species. Lastly, no model identifies the coding transcripts correctly in a single dataset, primarily due to its large proportions. On the other hand, ncRNAs have been correctly recognized by several models in all CNIT datasets except ZM and a few models in LG-OS, RP-AMT, RP-CS and RP-RC datasets. Most of these datasets consist of <500 ncRNAs. Thus, a 100% detection rate does not guarantee robustness and generalization of tools, especially when the disparity of classes is huge. The maximum detection rates of ncRNA-only PP-CA and PP-lOS datasets are 92% and 81%.

The ranks of the models on two datasets of fungi are presented in Figure 3I. These datasets belong to *S. cerevisiae* and have identical ncRNAs. The obtained *P*-value (2 × 10^−4^) is less than the assumed significance level and shows that differences in performance are significant. Overall, the results signify high performance with EU training source, lower performance with alignment-augmented models and no generalized consensus due to small datasets. LF-EU outperformed all other models and attained the top rank, but the following models are also very close, which indicates no superiority among models. The EU training set which also consists of *S. cerevisiae* instances helps to attain better performance than other sources. This finding also validates the cross-kingdom RNA diversity limiting the capabilities of models developed with species- and plant-level datasets. In terms of alignment-augmented models, only the PP-EU model makes it into the top 10 while others underperformed. This finding agrees with the analysis of the plant kingdom where absence of homology augmentation yields inferior models. Also, several models have identified ncRNAs correctly in both datasets, but all failed to do so for mRNAs (Figure 3J). The ncRNAs are identified correctly owing to their small number (613) in contrast to mRNAs. The IR is ∼16 for one dataset (Figure 3M), and its effect is observed on the PLN-EU model where precision, ncRNA FSC and MCC rates are low besides having high accuracy. There are a few exceptions in the outcomes; for example, the FL-AT model achieved a good performance without prior knowledge about the species. The LD tool whose performance is consistently low in previous analysis, ranked in the top 10 with the HS model. Thus, the comparative analysis of two datasets is insufficient to generalize the findings for the fungi kingdom.

### Benchmarking models on integrated datasets

The benchmark also comprises six heterogeneous datasets that have been constructed at kingdom (plants and a combination of animal, plants and fungi), phylum (vertebrate and invertebrate) and class (Mammals) levels. The ranks are presented in Figure 3K with significant differences in performances (*P* = 7 × 10^−19^). Refer to Supplementary File S7 for all results. Overall, the outcomes show high accuracy with the EU training source, good performance by alignment-augmented models and lower performance with AT- and PL-based models. Several tools attained excellent performance when trained with the EU training set. This illustrates the advantages of supplying heterogeneous over homogeneous data to ML algorithms to obtain generalized models. As a result, the performances of the top four models (RS, PP, PLN and LF) are high, with minor differences in accuracy as shown in Figure 3L. The remaining EU-based models also outshine their counterparts. The two alignment-augmented models, PP-EU and LAD-HS, also secured ranks in the top 10 while others (PP-HS, PP-AT, PP-PL and LAD-HSp) are significantly behind. PP-HS, PP-AT and LAD-HSp models are developed with species-level datasets and thereby fail to address the cross-species variations in RNAs. The PP-PL model underfits due to the lower representation of plant species in databases. As a result, models fed with PL training data always lag behind those fed with EU data. The class imbalance is high in these datasets as shown in Figure 3M. That is why models are highly accurate (>92%) despite low ncRNA precision (<85%), FSC (<85%) and MCC (<83%) rates. The high IR also reflects the bias in predictions, especially misclassifying coding transcripts as non-coding transcripts. All models trained with HS are biased towards ncRNAs, excluding RS-HS (Figure 3L). The dominance of animal RNAs suppresses the performance of AT- and PL-based models on these datasets, confirming the previous findings of low cross-species performance.

### Overlapping of *H. sapiens* training and test sets complicates the comparative analysis

The *H. sapiens* transcriptome has been used in RNA classification to develop most of the tools. The extensive research on humans provides researchers with enough data to build and compare the models to prove their effectiveness and efficiency. However, discovery of new transcripts and corrections in its annotations with more supporting information over the years has compelled researchers to select the training and test instances randomly. This approach complicates the comparative analysis at this species level as performance becomes sensitive due to common instances in training and test sets across tools. Thus, we test the hypothesis of whether variations in training data make the models robust or not for the same species.

The probability of overlapping between the training and test set is directly proportional to the size of the training set. The larger the training sets, the higher the overlap with validation sets. Such overlapping usually complements the performance with a better fit on the test dataset. To determine the impact of overlapping on the classification performance of HS, we compared nine training sets with the 18 benchmark sets. These training sets had been utilized in 13 tools, as shown in Figure 2B, and were chosen randomly while building the models. In these sets, PLEK and CPP tools have used the large training sets (45 000 and 57 000 instances) while others relied on small sets (<32 000 instances). Some tools are not compared due to unavailability of the training sets in the public domain (16,38), random selection of transcripts (33), a combination of multiple training sources (12) and development of models for plants only (34). Among these tools, large training sets have been utilized in LR (42000), LAD (>50 000) and RS (union of the FL, CPC2 and MR sets).

PLEK trained with a human set identified most mRNAs and ncRNAs correctly due to shared instances with many validation tests. As a result, the PLEK-HS model performed better than its other three models. It has an mRNA detection rate >88% for CPAT, CPC2, LGC, CPP-HS and LF datasets, but 50–80% for the remaining datasets. The ncRNA detection rate is also >98% on 13 datasets including 100% on mRNN HSC and HSC500 datasets. Even though model rank is close to half of total models in terms of FSC, it ranked sixth in ncRNA detection. These findings establish the correlation between high classification performance and the overlapping ratio for both classes in PLEK. In the CPP-HS model, where high overlapping is observed for ncRNAs only, the detection rate is >96% for all datasets, with 97.70% average recall rate. In contrast, only 69.13% of mRNAs are detected with this model. It ranked eighth in ncRNA detection but showed average performance overall (25th rank) Other training datasets with high similarity of ncRNAs belong to CPAT and CPC2 tools that also share almost identical sets. In terms of performance, CPAT identified mRNAs and ncRNAs almost equally; however, CPC2 has detected more ncRNAs. The balanced performance of the CPAT model is because of its optimized cut-off score, whereas CPC2 became biased towards ncRNAs because it relied on a default cut-off score of 0.5. Here, overlapping ncRNAs also complement the detection rate. In contrast, this effect is not observable for the remaining tools due to their small-sized training sets. These outcomes validate the overfitting of validation sets with the large training set and corroborate the previous findings (25,26,32).

Thus, developing and testing the performance of tools on the human species complicates the comparative analysis. An average performing model trained with a large training set can outperform a good model with a small training set when comparing them on the same species. Moreover, inferring from the present state of the AI domain where DL algorithms require huge training sets to develop superior and generalized models, the human species is a poor choice for building the models. The current approach will result in more overfitting of models only and therefore should be avoided.

### Non-coding RNAs are harder to identify than coding RNAs

**Figure 4.**
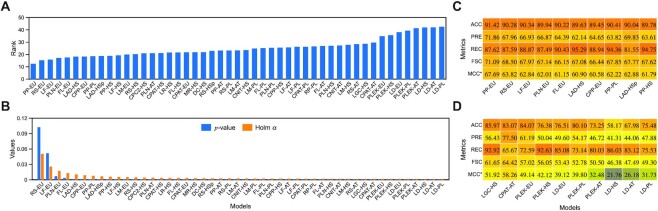
Statistical analysis of models on all datasets based on the F1-score and outcomes of the 10 top and 10 worst performing models. (**A**) Friedman ranks, (**B**) Holm post-hoc test, (**C**) prediction results of the top performing models and (**D**) prediction results of the worst performing models. MCC* indicates the mean of two-class datasets only.

We assessed the potential of the current tools to generalize the task of RNA characterization by carrying out the analysis on the whole benchmark as shown in Figure 4. The capabilities of tools are assessed in terms of classifying RNAs, recognizing ncRNAs and determining their susceptibility towards either class. Initially, statistical analysis of models is performed with Friedman and Holm post-hoc tests. The Friedman test is used to determine the differences in performance (Figure 4A) whereas the Holm post-hoc procedure helps to identify the best models for the classification of RNAs (Figure 4B). The obtained *P*-value of the Friedman test is 0 due to the large number of datasets and shows that the models involved in the benchmark produce significantly different outcomes. However, high ranks of models, between 12 and 43, depict the discrepancies in the prediction of RNAs across multiple species. The current models cannot sustain their performance on a large scale. The additional analysis with a post-hoc test shows that the control model (PP-EU) performs statistically better than all models other than RS-EU and LF-EU. The outcomes of the 10 top and 10 worst performing models provided in Figure 4C and D illustrate the differences in performances. Nevertheless, current tools are incapable of differentiating classes correctly as best models misclassified >8% RNAs. Thus, room for improvement is still substantial.

The obtained ranks show the prominence of integrated training sets and better performance with the augmentation of models with a similarity search. The models trained with the EU dataset have attained accuracy above 90%, including sequence-based RS, LF, PLN, FL and CPP tools and alignment-augmented PP and LAD tools. In contrast, models trained with HS and retrained with AT and PL produce inferior performances. Overexploitation of a particular species to solve the problem limited the capabilities of current models. When species-specific RNAs are used to build a model, the performances are likely to be suboptimal. In similarity-based augmentation, three models of PP (EU, HS and PL) and both models of LAD attained top ranks. Their high ranks confirm the benefits of relying on the databases to distinguish mRNA and ncRNA, primarily when species are known. However, a similarity search cannot be exaggerated as the most relevant descriptor. Instead, it helps to identify RNA more accurately with the available homologue information. These observations are similar to our previously discussed findings.

The outcomes of the worst models (ascending order) presented in Figure 4D have ranks between 29 and 43, and belong to LGC, CPAT, PLEK and LD tools. All four models of PLEK and LD achieved higher ranks in the benchmark. In the case of PLEK, the visual plots (as shown in Figure 2C) corroborate the findings as significant overlap of mRNAs and ncRNAs can be observed. LD also has a low performance as reported in existing studies (11,16,32). The mean differences in the accuracy of the best and worst models are >5% and reach as high as 33%. In terms of the impact of training sources on PLEK and LD performances, EU is the winner.

Secondly, we assessed all instances in the benchmark to identify which class is tough to recognize. There are 3.4 million transcripts in the benchmark, representing an ∼83% and ∼17% share of mRNA and ncRNA, respectively. Out of the total transcripts, ∼80 000 mRNAs and 77 000 ncRNAs are misclassified with the best performing model (PP-EU). These misclassifications represent a significant share of ncRNAs (13.6%) but a tiny share of mRNAs (0.03%). The FSC and MCC rates of <72% and 68% also show greater complexity of detecting ncRNAs. By extrapolating the misclassification of ncRNAs with respect to mRNAs that are five times higher in the benchmark, the numbers would be close to 400 000. In contrast, the maximum misclassifications of mRNAs in the top models (i.e. PP-HS) are 267 000. Therefore, it can be established that the ncRNAs are more challenging to identify than the mRNAs. This limits the practical applications of current tools to annotate the transcripts since ncRNAs’ share has been rising, and they will eventually dominate the transcriptome landscape. Thus, it is necessary to identify the ncRNAs correctly as compared with mRNAs, and, therefore, the tools should have better capabilities to recognize them.

Lastly, detecting ncRNAs correctly with the current tools is still problematic (FSC and MCC <72%), and their performance varies across species. The top four models have <90% recall and <65% precision, as shown in Figure 4C. On the other hand, FL-EU, LAD-HS, PP-PL and PP-HS models detected up to 95% ncRNAs but have low precision. Thus, some models are also biased and can easily misclassify the mRNAs as ncRNAs.

### Hard examples and the possibility of false alarms

The misclassifications of RNAs pose another challenge for biology and ML communities, i.e. whether misclassifications represent hard examples or incorrect annotations. The latter option is expected in the biology domain where , for instance, >2000 human and mouse coding genes have been reclassified as non-coding in the GENCODE database since their initial release (51). Unlike conventional classification problems such as image classification, text classification, lesion detection, content-based image retrieval, stock market prediction, and so on where such errors can be corrected with high accuracy by experts, RNAs require extensive wet lab and transcriptomic assembly experiments for validation. Such corrections are prevalent in many biological studies due to the limitations of current sequencing technologies, time-intensive laboratory experiments and high cost. In RNA classification, false positives and negatives occur when mRNAs are labelled as ncRNAs, and vice versa. Several factors are responsible for the inaccurate labelling of RNAs. The false positives occur due to the partial recovery of transcripts from *de novo* assembly and inability of current sequencing technologies to detect small ORFs in transcripts. In contrast, false negatives occur owing to the presence of ORFs in ncRNAs, alternative splicing of transcripts and pseudogenes. As the current knowledge about quantity, quality, classes and evolutionary conservation of RNA transcripts is limited, the possibilities of errors are high. The validation of RNA type usually requires evidence from several sources such as experimental data published in the literature, performing large-scale mass spectrometry experiments, examining the sequence similarity to orthologues with other species, searching for sequence homology in the Pfam domain (52,53) and ribosome profiling to detect ORFs, especially smaller ones (54).

In a nutshell, this problem cannot reap the benefits of rectifying mistakes by observing the instances directly, as followed in traditional applications. The false-positive and negative instances could hamper the generalization capabilities of the ML algorithms and may produce incorrect annotations with poor fit of training data. Nonetheless, ML and DL approaches have emerged as the frontrunner to solve the related problems such as false-positive discovery in protein function annotation (55), somatic variant refinement (56), genome-wide association studies (57) and homology detection (58).

**Figure 5.**
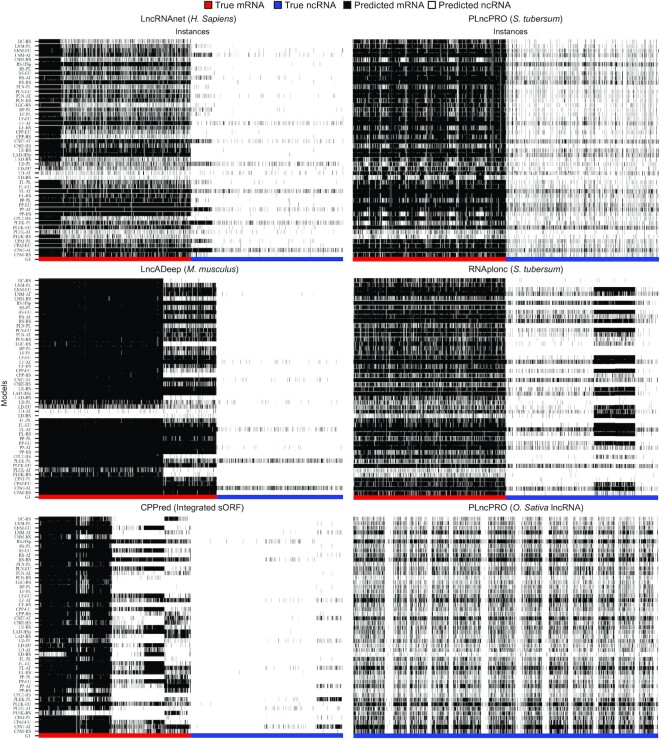
Classification decisions of models at the instance level of six datasets to illustrate the possibility of false positives and negatives. *H. sapiens* from LncRNAnet, *M. musculus* from LncADeep and integrated small ORFs from CPPred datasets show the majority of mRNA misclassifications, whereas *S. tuberosum* from PLncPRO and RNAplonc as well as *O. sativa* from PLncPRO demonstrate the inaccuracies prevalent in ncRNA identification.

To demonstrate the issue of incorrect annotations in the benchmark, the challenging instances are analysed. These are the protein-coding and non-coding transcripts that most models have misclassified consistently. We have selected those datasets where such transcripts occur frequently and presented the prediction of all models at the instance level in Figure 5. For each RNA type, three datasets have been selected to illustrate the misclassifications. These transcripts are either hard examples or false alarms. Analysing the predictions instance-wise, white lines in mRNAs and black lines in ncRNAs indicate the poor performance of models to distinguish transcripts. Among these, hard instances could be consistently misclassified mRNAs and ncRNAs with the majority of models. In contrast, several columns, completely white or black but in another class, provide strong evidence of incorrect annotations. The false positives could be black lines in the blue area, whereas white lines in the red area could be false negatives. In LR-HS and LAD-MM datasets, mRNAs are incorrecly classified. Likewise, the CPP-sEU dataset contains small ORF transcripts that are difficult to identify. In contrast, PP-ST and RP-ST datasets have several instances of misclassified RNA, but mostly ncRNAs. Most tools have predicted mRNAs in the lncRNA-only PP-IOS dataset. This problem has also been observed in other datasets (see Supplementary Figure S2). Certainly, the probability of false alarms cannot be overlooked for such instances. In addition, the problem of under- and overfitting can be validated from these plots as several models are biased towards either class. Specifically, AT- and PL-based models are biased towards mRNAs on the LR-HS dataset, whereas both CPAT-AT and PLEK-PL models predicted most RNAs as coding on the LAD-MM dataset. In both *Solanum tuberosum* datasets, several models are biased towards mRNAs. On the other hand, the LD-HS model predicted most RNAs as non-coding in all six datasets. Likewise, LD-AT and PLEK-HS models leaned towards ncRNAs on LR-HS and CPP-sEU datasets.

**Figure 6.**
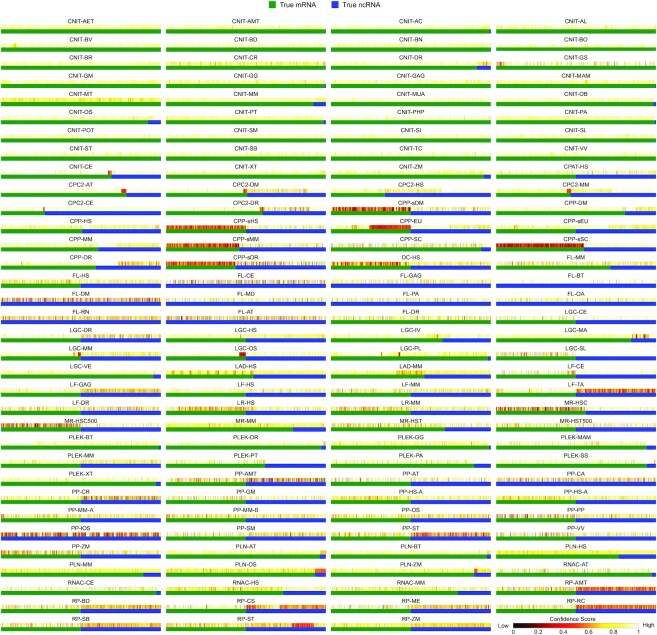
The average confidence scores of models on the benchmark.

Furthermore, we have averaged the classification decisions of all models for each dataset to analyse their common consensus. This not only helps to identify hard instances, including false alarms, but also builds a benchmark to validate the performances of existing and new tools. Figure 6 presents the mean scores on the benchmark where visual inspection reveals low confidence of many trancripts in both classes. In particular, this problem occurs with datasets belonging to plant species from PP, RP and LF sources and have small RNA transcripts (CPP-sDM, CPP-sHS, CPP-sMM, CPP-sSC, CPP-sDR and DC-HS). A few instances from CPP, MR and LGC testing sets are also tough to classify. The chances of false alarms are high as the benchmark comprises a dataset that is almost a decade old. For instance, GENCODE reported the collection of ∼33 000 and 22 000 novel and updated mRNAs and lncRNAs transcripts from 2018 to 2021 (51). These findings also highlight the dynamic nature of the problem arising from the continuous revisions of databases that are still ongoing. These instances will be of interest to ML researchers for solving this problem with better computational approaches and to biologists for discovering the characteristics of sophisticated RNAs.

### Constructing a new challenging classification dataset

We constructed a new RNA classification dataset from the confidence score of accurate recognition of transcripts by 48 models from 17 tools. We named this dataset RNAChallenge, in which transcripts correctly recognized by at most eight models have been selected from the benchmark. After removing the duplicate sequences with the CD-HIT tool, 27 283 transcripts were obtained. This test set contains 16 243 mRNAs and 11 040 ncRNAs belonging to species from animal, plant and fungi kingdoms (Supplementary File S8). We assessed the performance of 24 tools providing 58 models on this dataset and present the outcomes in Table 1. Overall, the current tools show poor performance, with accuracy <50%, ncRNA precision <40% ncRNA recall <71%, FSC <47% and MCC <0 rates. No model is able to identify even half of the RNAs. The negative MCC rate signifies the substantial under- and overfitting of the challenge dataset as current models skewed towards either mRNAs (very low recall) or ncRNAs (recall exceeds accuracy). As a result, inferior tools such as PLEK and LD attained higher classification performance than PP, LAD, RS and LF tools that exhibit superior performance on the benchmark.

**Table 1.** Performance of tools on the RNAChallenge dataset

Models	ACC	PRE	REC	FSC	MCC	Models	ACC	PRE	REC	FSC	MCC
**CPAT-HS**	5.04	0.29	0.40	0.34	–90.16	**BI-HS**	27.32	10.76	10.91	10.84	−50.49
**CPAT-AT**	15.46	0	0	0	−73.18	**CRE-AT**	47.00	1.06	0.34	0.51	−30.77
**CPAT-PL**	1.85	0.01	0.01	0.01	−96.25	**CNIT-HS**	6.46	8.06	12.60	9.83	−86.66
**CPAT-EU**	3.78	1.36	1.93	1.60	−92.32	**CNIT-AT**	10.36	0.65	0.80	0.71	−80.84
**PLEK-HS**	11.97	13.85	22.51	17.15	−75.19	**CPP-HS**	5.11	6.50	10.05	7.89	−89.46
**PLEK-AT**	19.49	19.04	30.43	23.42	−59.06	**CPP-EU**	6.33	1.50	2.04	1.73	−87.49
**PLEK-PL**	11.66	6.08	8.19	6.98	−76.68	**LF-HS**	0.65	0.41	0.61	0.49	−98.66
**PLEK-EU**	21.15	4.44	4.62	4.53	−62.62	**LF-AT**	10.11	0.64	0.80	0.71	−81.25
**LI-HS**	2.91	4.41	6.78	5.35	−94.03	**LF-PL**	0.76	0.29	0.42	0.34	−98.43
**LI-AT**	10.90	5.99	8.18	6.92	−78.04	**LF-EU**	2.54	1.04	1.49	1.23	−94.78
**LI-PL**	3.13	4.64	7.13	5.62	−93.58	**RP-AT**	2.50	2.09	3.07	2.49	−94.82
**LI-EU**	10.78	3.56	4.62	4.02	−79.04	**LGC-HS**	4.00	5.01	7.64	6.05	−91.72
**CPC2-HS**	0.83	1.30	1.93	1.55	−98.29	**PGF-HS**	20.65	0.48	0.46	0.47	−65.49
**PP-HS**	8.78	6.61	9.55	7.81	−81.88	**PLN-HS**	7.65	7.45	11.23	8.96	−84.06
**PP-AT**	13.88	0.67	0.76	0.71	−75.24	**PLN-AT**	5.07	2.44	3.45	2.86	−89.66
**PP-PL**	3.12	2.57	3.78	3.06	−93.54	**PLN-PL**	2.47	3.96	6.07	4.79	−94.94
**PP-EU**	10.60	0.87	1.07	0.96	−80.36	**PLN-EU**	6.40	2.38	3.29	2.76	−87.16
**FL-HS**	11.35	2.11	2.63	2.34	−78.63	**RS-HS**	15.76	0.77	0.84	0.80	−72.36
**FL-AT**	21.41	0.68	0.65	0.67	−64.34	**RS-AT**	5.45	2.16	3.02	2.52	−88.98
**FL-PL**	2.58	1.38	2.00	1.64	−94.68	**RS-PL**	1.04	1.67	2.49	2.00	−97.84
**FL-EU**	5.57	3.56	5.12	4.20	−88.54	**RS-EU**	5.89	2.22	3.08	2.58	−88.14
**LD-HS**	33.67	34.37	70.30	46.17	−27.20	**RS-HSp**	14.54	0.28	0.31	0.29	−74.43
**LD-AT**	25.15	24.67	41.38	30.91	−46.77	**LMD-HS**	6.51	0.38	0.51	0.44	−87.49
**LD-PL**	21.02	17.86	26.44	21.32	−56.31	**NCR-HS**	48.99	39.25	47.55	43.00	−2.43
**LD-EU**	19.45	20.08	33.25	25.04	−59.17	**LM-HS**	1.72	2.70	4.08	3.25	−96.46
**LAD-HS**	9.16	7.26	10.58	8.61	−81.04	**LM-AT**	5.36	0	0	0	−89.64
**LAD-HSp**	16.68	1.18	1.28	1.23	−70.79	**LM-PL**	0.65	0.22	0.32	0.26	−98.66
**MR-HS**	13.68	1.06	1.23	1.14	−75.37	**LM-EU**	5.96	1.27	1.73	1.47	−88.23
**LR-HS**	10.20	1.72	2.17	1.92	−80.68	**DC-HS**	4.50	5.87	9.04	7.12	−90.72

The six tools, LI, BS, CRE, PGF, LMD and NCR, used for testing purposes also exhibit similar outcomes. LI has four models, CRE has only the AT model, and BS, PGF, LMD and NCR tools have only the HS model (Figure 2A). To ascertain whether high performance of some of these tools generalizes, additional experiments are performed with a sample of six datasets from the benchmark (see Supplementary Table S1). The outcomes of models are analogous for 18 tools, i.e. (i) alignment-augmented PL models producing lower performance; (ii) the EU training dataset yielding higher performance; (iii) AT- and PL-based models having low performance; and (iv) best performers on the RNAChallenge test set lacking in the benchmark, and vice versa. The CRE tool utilized the Uniprot database to complement the performance with sequence search, but failed to achieve reasonable performance on sample datasets. The AT- and PL-based models of LI lag behind its HS and EU models. This supports the previous findings about the superiority of heterogeneous sources and failure of AT- and PL-based models to capture the transcriptome diversity of the wide range of species. The HS-based models of the remaining tools show higher misclassifications for plant species. On integrated and animal species datasets, PGF and LMD showed good performance while the NCR tool emerged as the least accurate. However, it attained the best performance on the RNAChallenge dataset while significantly underfitting sample datasets.

**Figure 7.**
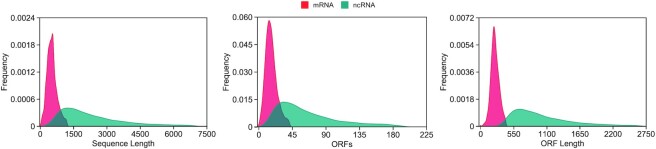
Distribution of sequence length as well as ORFs and their length in the RNAChallenge dataset.

Further, we analysed the characteristics of these RNAs, such as sequence length and ORFs, to find out the reasons for higher misclassifications. In particular, distributions of sequence length, ORFs and ORF length in mRNAs and ncRNAs are measured, and the outcomes are presented in Figure 7 after removing the outliers (see Supplementary Figure S3 for results on all sequences). The ORF Finder tool (NCBI) is used to search for ORFs that begin with the ‘ATG’ codon and have a minimum length of 30. This codon is selected because it has been widely used in coding potential tools, and the minimum length ensures small RNA coverage. Overall, protein-coding RNAs of this dataset are shorter in length and have fewer and smaller ORFs than ncRNAs. Specifically, the mRNA subset has 22 sequences without a ORF, 286 sequences with ORFs <100 nt and >13 000 sequences (∼80%) with ORFs <300 nt. Thus, small ORFs caused the incorrect identification of mRNAs as ncRNAs. On the other hand, all ncRNAs have at least one ORF of >300 nt size which results in misclassifications of ncRNA as mRNA by most models. In addition, these findings suggest that the selected transcripts have different distributions in the feature space than classification boundaries learned with the most accurate tools. As a result, underperformers tend to overfit while the best models underfit the RNAChallenge dataset. To sum up, this dataset will present a challenge for new tools to validate their generalization capabilities to differentiate mRNAs and ncRNAs.

### Machine learning approaches outperform deep learning

The last decade in AI research has observed a paradigm shift from feature engineering to learning through DL. The feature engineering approach, commonly referred to as handcrafted features, involves designing the features by exploring certain traits of the investigated problem. On the other hand, automated approaches aim to discover the abstract representations of problems by directly learning from the data. These representations are usually hierarchical where higher levels are defined in terms of lower ones. The high-level features have shown high tolerance to variations in training sets and outstanding performance in solving numerous real-world problems (59). In the biology domain, DL approaches surpassed conventional techniques in solving protein folding (60), DNA- and RNA-binding proteins (61), protein function prediction (62), transcription factors (63) and peptide–protein interaction (64) problems.

In RNA classification, handcrafted features have been utilized extensively, as shown in Figure 2A, where all descriptors are manually designed except SeqEE. Only two methods, MR and LR, are end-to-end DL frameworks that have used the sequence encoding and embedding mechanisms to extract the feature automatically. Three other methods have used some aspects of DL but rely on handcrafted features too. LAD used a deep belief network whereas DC used the convolutional layers on the manually extracted features. RS relies on IGLOO layers built using the 1D convolutional layer and handcrafted features such as *k*-mers, ORF length and amino acid frequencies. Similarly, LMD relied on CNN-based features from sequences besides the ORF, Fickett, hexamer and *k*-mers.

The end-to-end DL tools were inferior in performance by large margins to ML methods, as observed from the outcomes (Figures 3 and 4). Also, the tools utilizing DL techniques with manual features have shown much better performance and outperformed end-to-end models across all domain-level experiments. The human model of MR and LR attained good performance with ranking in the top 10 for human species but have shown low performance for the remaining cases. Overall, MR obtained the 20th rank with 88.84% accuracy, ∼81% ncRNA detection and <68% precision rates. LR attained the 17th rank with 89.26% accuracy, >84% recall and ∼65% precision. Undoubtedly, highly accurate DL models for the RNA classification problem are yet to be seen in this domain, and it opens up new opportunities to develop better tools.

## DISCUSSION

The systematic analysis of the coding and non-coding classification problem on a large-scale benchmark helped to identify the crucial factors affecting the performance of current tools. To begin with, the evolutionary pressure on species has significant implications for the diversity of RNAs (65–67). As species undergo different rates of evolution in different lineages, this reduces the uniformity of both classes. Accordingly, different types of RNAs are evolving at a varying rate. For example, mRNAs and miRNAs are more conserved than lncRNAs in mammals and vertebrates because they evolved more slowly (66). Similarly, evolution at the organ level is non-uniform; protein-coding transcripts of the brain are less susceptible to sequence evolution than those of the testis (66). Thus, substitution rates in sequences that vary from RNA types to organs complicate the classification task and pose a significant challenge in developing generalized models. Another crucial issue is the imbalance of coding and non-coding transcriptome data. Although it is an intrinsic property of the RNA world, disagreement between the knowledgebase and current landscape demands continuous attention to address this issue while building models. Ideally, ncRNAs should dominate due to their larger share and smaller size in genomes; however, the technical limitations to detect them together with protein-centred research made mRNAs the majority class. As a result, models are often biased towards mRNAs, but a substantial shift in the share of classes could decrease their performance over time because ncRNAs will exceed them eventually. So, models should be fine-tuned regularly to incorporate the latest data.

The absence of gold standard training sets to build and evaluate the tools is a major concern that causes difficulties in determining the true performance of tools. From the findings, it is evident that selecting training data from multiple species is beneficial to develop the models rather than relying on a particular species only. So, the use of species-specific models should be avoided, and high-quality transcripts from heterogeneous sources should be selected to build a single model that can annotate a wide range of species. A gold standard dataset can be constructed through experimentally validated mRNAs and ncRNAs from multiple species while maintaining their equal representation. The transcripts can be obtained from high-quality databases built for specific RNAs, namely mRNAs (46,47,51,68), miRNAs (69), tRNAs (70), piRNAs (71), snoRNAs (72) and circular RNAs (73), as well as other popular sources (74,75). Additionally, the performance of tools is directly proportional to in-depth research conducted on the species. More comprehensive exploration yields high quality and large amounts of data for building better models, and vice versa. For example, animal species have been explored more extensively than other kingdoms such as plants and fungi, and therefore are expected to be classified more accurately. While mRNAs in animal and plant species have been largely detected, ncRNAs remain highly under-represented in plants. Thus, sufficient information to differentiate RNAs could be obtained by building models with a broad range of species.

Aligning sequences against the known databases helps to boost the performance as validated from the higher classification accuracy of PP and LAD tools, but it comes at an additional computation cost. Here, improved search and DL techniques can be employed to cope with this issue. The recently developed alignment tools such as DIAMOND (45), RAPsearch2 (76) and MMseqs2 (77) are far more efficient than conventional BLAST tools. Likewise, DL models are also being developed to detect the homologues directly (78–81), and are also efficient. For instance, ProtTrans (80) is faster than the MMseqs2, ESM (78) achieved performance close that of to hidden Markov model- (HMM) based tools and ProtCNN (82) outperformed the *blastp and phmmer* tools in a remote homology detection task. Thus, the available knowledgebase of proteins that complement the model performance always should be included in developing new tools.

Identification of false alarms is crucial, as erroneous annotations could have a profound negative impact on the performance of tools. This problem will become more apparent with the detection of more transcripts and as coverage of species grows. An elegant example of this case is small RNAs that have been considered mainly non-coding due to the limitations of computational and experimental approaches to detect small ORFs (83). So, inaccuracies in training sets may cause under- and overfitting of models and conceal their actual performance in real-world scenarios. To deal with this problem, regular benchmarking of high performance computational tools could provide better confidence scores to categorize RNAs. The hard instances could be tested experimentally for validation, whereas easy ones could be used for annotations.

### Conclusion

A large-scale study of coding potential prediction tools offers new insights to deal with a complex classification problem. The challenges for building the models that arise from the dynamic nature of the problem have been highlighted and, subsequently, a new test set serving dual goals has been presented. It includes comparing the performances of newer *in silico* methodologies for obtaining robust models and validating them experimentally to identify the potential false RNAs, novel characteristics occurring from exceptional cases and bifunctional RNAs. Overall, the benchmarking results are encouraging, but there is substantial room for improvements to develop accurate and reliable general-purpose tools. In conclusion, we expect ML to be a useful RNA identification tool in the meantime. Future tools could focus on end-to-end DL models to find better solutions without relying on handcrafted features. The combination of supervised and unsupervised learning-based DL approaches that have shown significant improvements in other biology domains is another option that can resolve the performance bottlenecks in predicting the coding potential of transcripts.

## DATA AVAILABILITY

All datasets utilized in this study were collected from the existing tools only. The publicly available links to download datasets and tools are listed in Supplementary File S9. All other data supporting the findings of this work are available within the paper and its supplementary files. The RNAChallenge dataset is available at https://github.com/cbl-nabi/RNAChallenge.

## Supplementary Material

gkac1092_Supplemental_Files
