# Supplementary material for: A large-scale benchmark study of tools for the classification of protein-coding and non-coding RNAs
Source: Nucleic Acids Res. 2022 Nov 24;50(21):12094–111. doi: 10.1093/nar/gkac1092 (PMC9757047; doi:10.1093/nar/gkac1092)
Supplement: gkac1092_Supplemental_Files [file gkac1092_supplemental_files.zip › Captions.docx]

Captions of supplementary files

| **File Name** | **Caption** |
| --- | --- |
| Supplementary File 1 | Details of benchmarking datasets |
| Supplementary File 2 | Prediction outcomes on *H. sapiens* datasets |
| Supplementary File 3 | Prediction outcomes on *A. thaliana* datasets |
| Supplementary File 4 | Prediction outcomes on animal datasets except for *H. sapiens* |
| Supplementary File 5 | Prediction outcomes on plant datasets except for *A. thaliana* |
| Supplementary File 6 | Prediction outcomes on Fungi datasets |
| Supplementary File 7 | Prediction outcomes on Heterogeneous datasets |
| Supplementary File 8 | RNAChallenge dataset construction by selecting correctly recognized RNAs by at most 8 models from the benchmark |
| Supplementary File 9 | Publicly available links to download  datasets and tools |
