# Supplementary material for: A large-scale benchmark study of tools for the classification of protein-coding and non-coding RNAs
Source: Nucleic Acids Res. 2022 Nov 24;50(21):12094–111. doi: 10.1093/nar/gkac1092 (PMC9757047; doi:10.1093/nar/gkac1092)
Supplement: gkac1092_Supplemental_Files [file gkac1092_supplemental_files.zip › supp.pdf]

Supplementary Material: A large scale benchmark study of tools for the classification of protein-coding and non-coding RNAs

Dalwinder Singh and Joy Roy

1 Materials

Fig. S1 presents the length distributions of validation datasets belonging to species, subphylum, class, kingdom and domain. The benchmark represents distribution of all datasets used in the study. The length distributions of mRNAs are provided in Fig. S1A whereas ncRNAs length distribution is shown in Fig. S1B. The plots indicate the presence of outliers in both classes.

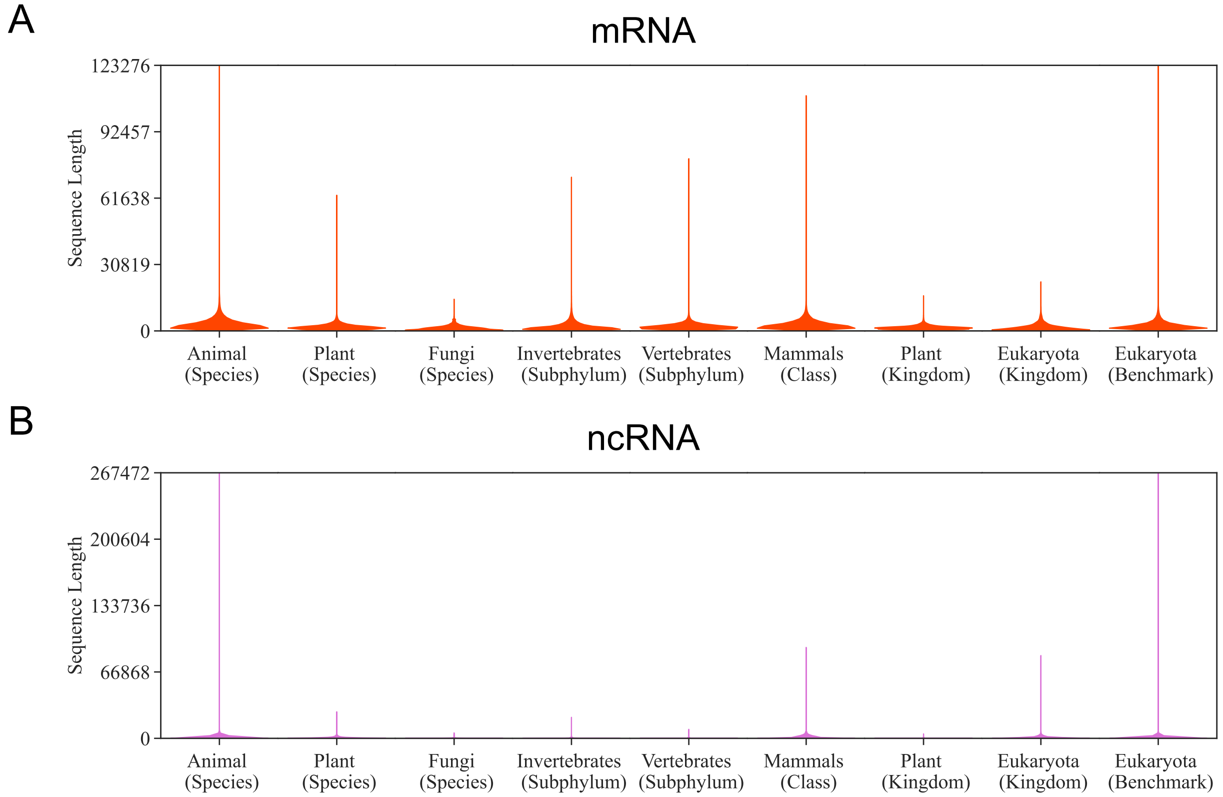

Figure S1: The distribution of sequences length in validation datasets at species, subphylum, class, kingdom, domain and benchmark levels

2 Methods

The brief dicussion about the methods are provided as follows:

Coding Potential Assessment Tool (CPAT) [1] was an alignment-free method that used the ORF based features, Fickett and hexamer scores to differentiate the mRNAs and ncRNAs. The training was performed using logistic regression (LR) classifier on a class balanced dataset comprising human transcripts. The pre-trained models for other animal species such as mouse, fly and zebrafish are also available; however, experiments are performed using a human model and three newly constructed models.

The predictor of lncRNAs and mRNAs based on an improved k-mer scheme (PLEK) [2] performed classification without sequence alignment. The conventional k-mer scheme was calibrated with a factor relating to the length of the pattern for obtaining a feature vector. The training is performed using SVM with radial basis function (RBF) kernel on a balanced human dataset where C and γ parameters of classifiers were optimized using grid search. The pre-trained human model and three new models are built for experiments.

Lncident [3] aimed to identify RNAs more efficiently while maintaining the classification performance. To achieve efficiency, features were extracted from the ORF regions of sequences only, including the k-mers descriptors. The models were constructed species-wise (Human, mouse, and *C. elegans*) using the SVM-RBF classifier. In the benchmark, we used the human model trained with class balanced dataset and also constructed three models for comparative analysis.

Coding Potential Calculator (CPC) 2 [4] was the improved version of the CPC [5] in terms of accuracy and efficiency. It is an alignment-free tool that requires Fickett score, ORF descriptors, and isoelectric point (pI) feature to classify mRNAs and ncRNAs. A sole SVM based model was developed using the RBF kernel on the imbalanced human dataset containing $\sim$1.71 times more mRNAs than lncRNAs. We have used the human model for evaluating validation datasets from this tool.

The Plant Long Non-Coding RNA Prediction by Random forest (PLncPRO) [6] was primarily developed for RNA characterization of plant species. This tool relied on the alignment of sequences using BLASTX and other alginment-free descriptors such as transcript length, ORF, and tri-mers to discriminate the mRNAs and lncRNAs. The experiments were conducted in several ways on balanced datasets using Random Forests classifier. In this benchmark, the performance of PLncPRO is assessed by constructing human model from the supplied data and three models from standard training datasets. To detect the sequence homology, we used the latest UNIPROT database.

The flexible extraction of lncRNAs (FEElnc) [7] is an alignment-free tool to separate mRNAs and lncRNAs which used ORF, k-mers profiles, and sequence length descriptors. The training is performed on a class balanced human dataset using the RF classifier and the optimized cut-off was set through 10-fold cross-validation. We have built four models, similar to PLncPRO, by using a supplied human dataset and the other three standard training datasets.

longdist [8] was also an alignment-free tool that utilized ORF descriptors and k-mers (2, 3, and 4) reduced with Principal Component Analysis (PCA) to train the model using SVM-RBF classifier to distinguish mRNAs and lncRNAs. The learning was performed by optimizing the SVM parameters with a grid search scheme, and several species-specific and integrated models were developed. We used the pre-trained human models and also constructed three models for experimentation with this tool.

LncADeep [9] was another hybrid tool that used the combination of alignment-free and alignment-based descriptors for the classification of mRNAs and lncRNAs. ORF, Longest CDS (LCDS), hexamer score, UTR coverage, GC content, Fickett score and entropy density profile (EDP) were used as alignment-free descriptors. On the other hand, HMMER index computed by aligning the sequences against the Pfam database were alignment-based descriptors. This tool was developed for full-length as well as partial-length transcripts. In the first model, ORFs and UTR descriptors were computed from the full-length transcripts, whereas second model utilized the longest CDS from the longest ORF with missing 3' ends and hexamer-based CDS for features. The models were built with deep belief network (DBN) using class imbalanced human training datasets having mRNAs to lncRNA ratio of 1.41 and 3.35 for full, and the combination of full and partial length transcripts. To test the performance on the benchmark, both models have been used in the study.

mRNN [10] was an end-to-end deep learning method for distinguishing the mRNAs and lncRNAs. It did not require any sequence alignments and genomic descriptors for prediction. Instead, the RNA sequences were passed through the embedding layer to learn the patterns through gated recurrent units (GRU). The training was performed on the class balanced human data, and five models were tested individually and with an ensemble approach. This study uses an ensemble approach to assess the performances on the benchmark datasets due to its higher performance.

LncRNAnet [11] was another end-to-end deep learning approach that combined the convolution neural networks (CNN) and Long short-term memory (LSTM) for differentiating the lncRNAs from mRNAs. It is an alignment-free tool where CNN was used to recognize the ORF from the sequences and feature extraction, whereas LSTM learned the sequential patterns from the one-hot encoding of transcripts. A softmax classifier combined the ORF features and LSTM outputs to predict the RNA type. The training was performed on the class balanced human transcripts, and this pre-trained model is used for experiments on the benchmark.

The BiologicAl Sequences NETwork (BASiNET) [12] used the combination of graph theory and machine learning approaches to classify RNAs. The features were extracted by mapping the sequences to an undirected weighted network and calculating its topological characteristics. The training was performed with decision trees, and training and testing datasets were obtained from PLEK and CPC2 tools. We have used the default human model provided with the tool.

CREMA [13] was developed to characterize mRNAs and lncRNAs from plant species. It relied on both alignment-based and free descriptors to construct the feature set. The sequences were aligned with SwissProt database using DIAMOND tool to detect homologs and extract features. In alignment-free descriptors, ORF, transcript length, GC content, Fickett score, hexamer score, transposable elements (TE) were used. The training was performed with gradient boosting and random forest approaches to construct the species dependent models with class-imbalanced datasets. We have selected *A. thaliana* based model for experiments, and have used the latest UNIPROT database to search sequences with the DIAMOND tool.

Coding-Non-Coding Identifying Tool (CNIT) [14] was a more accurate and efficient version of Coding-Non-Coding Index (CNCI) tool to classify mRNAs and ncRNAs. Similar to CNCI, this improved method also relied on the most-like CDS (MLCDS) regions arising from the ORF descriptors for differentiation and did not need to align the sequences. The training involved constructing human and *A. thaliana* model using class balanced datasets and XGBoost classifier. We have used both models to assess the performance on the benchmark.

The coding potential prediction (CPPred) [15] was an alignment-free method for the classification of mRNAs and ncRNAs. This method utilizes the ORF descriptors, isoelectric point, Instability, Gravy, Fickett score, hexamer score, and composition, transition, and distribution (CTD) descriptors. Two prediction models were built using the SVM-RBF classifier with an imbalanced human and Integrated datasets with the mRNA to ncRNA ratio of 1.38 and 1.9. We have selected both models for benchmark evaluation.

LncFinder [16] was an alignment free method for classifying coding and long coding RNAs. The extracted features include ORF, the distance between mRNAs and ncRNAs, free energy, multi-scale secondary structure-derived sequences, electron-ion inter-action pseudo-potential (EIIP) descriptors. The class balanced datasets were used to develop species specific models with SVM classifier. In this work, we used the pre-trained human model and constructed three models from standard training sources to evaluate the benchmark. It is worth mentioning that multi-scale secondary structure-derived features were omitted owing to their lower performance.

RNAplonc [17] was another plant based tool for distinguishing mRNAs and lncRNAs without sequence alignments. It used GC content, k-mer, ORF, and sequence length to learn the patterns with REPTree classifier. A balanced training dataset constructed from *A. thaliana*, *C. sativus*, *G. max*, *P. trichocarpa* and *O. sativa* species was used for training. This integrated plant model is considered for benchmark evaluation. It is worth mentioning that RNAplonc skips transcripts when an ORF is not found with *txCdsPredict* and therefore, we labeled such transcripts as lncRNA similar to previous works [18].

LGC [19] was an alignment free tool that utilized ORF descriptor and guanine - cytosine (GC) content to discriminate lncRNAs from mRNAs. The training was performed using the class imbalanced human dataset having mRNA to lncRNA ratio of 1.40 using maximum likelihood estimation. The human model of this tool is used for benchmarking.

PredLnc-GFStack [20] used the combination of feature selection and stacking approach to differentiate the coding and non-coding RNAs. It did not require alignment and the features were extracted from a wide range of sequences descriptors such as codons, ORF, GC, transcript length, k-mer, CTD, Hexamer score, Fickett score, UTR coverage, molecular weight, pI, GRAVY, instability index, EDP, and EIIP. The feature subsets were selected with a genetic algorithm using RF classifier. The stacked ensemble learning technique was used to build the final models with class-imbalanced datasets from human (mRNAs to ncRNAs ratio 1.28) and mouse species. Only human model is used to assess the performance of this tool on the benchmark.

The Predicting lncRNAs (PreLnc) [21] was an alignment free tool for discriminating the mRNAs from the lncRNAs using the sequence length, ORF descriptor, Fickett score, hexamer score, GC content, codons, isoelectric point, and tri-nucleotides subsets features. The training was performed on class balanced datasets from three animal and plant kingdom species using the RF classifier. Using this tool, four models are built to evaluate the benchmarking datasets.

RNAsamba [22] was a alignment free tool based on deep learning for the classification of mRNAs and ncRNAs. The whole RNA and ORF sequences were used to train the network with convolution based IGLOO layers. The protein sequence, k-mer and ORF descriptor from the ORF sequence were extracted and fed to dense layers. On the other hand, the whole sequence was fed to IGLOO layers directly. The outputs of whole and ORF sequence were combined through a weighting scheme to obtain the classifier. Learning was carried out with human full and partial length transcripts from existing works, and two models were built with imbalanced datasets. We constructed three additional models and used both human models to evaluate the performance on the benchmark.

lncRNA-Mdeep [23] was based on multimodal deep learning approach to distinguish mRNAs and lncRNAs. It aggregated the outputs of two DNN models and a CNN model for RNA classification. In one DNN model, ORF, Fickett and Hexamer (OFH) features were fed, whereas second model used k-mer features. In CNN model, the inputs were one-hot encoded sequences. The training was performed on the class-balanced human dataset, and only this model has been used for comparative analysis.

NCResNet [24] used the CNN to distinguish the mRNAs and ncRNAs with ORF, Fickett score, hexamer score, codons, GC content, distance between mRNAs and ncRNAs, CTD, instability index, GRAVY, pI, molecular weight and EIIP descriptors. A deep residual network was used to build the model with class-imbalanced human training set (mRNAs to ncRNAs ratio 1.28). Only this model is used in the benchmark evaluation.

LncMachine [25] was developed for plant based species originally to categorize mRNAs and lncRNAs. It did not require aligning sequences and used ORF, sequence length, GC content and pI descriptors for classification. The training was performed on the class balanced dataset and RF classifier. We have built four models with this tool to assess the performance of benchmarking datasets.

The deep neural network for coding potential prediction (DeepCPP) [26] was an alignment free tool for distinguishing mRNAs and ncRNAs. It utilized the ORF descriptors, hexamer score, Fickett score, k-mer and g-gap, and nucleotide bias features for the classification. CNN was used to train models belonging to human, vertebrate, and insect species with class imbalanced datasets (mRNAs to ncRNAs ratio of 1.38, 1.70, and 2.02). We have used the pre-trained human model from this toll for benchmarking purposes.

3 Hard examples and the possibility of false alarms

The prediction of all models on 17 additional datasets, as shown in Fig. S2, illustrates the misclassifications of mRNAs and ncRNAs. From the plots, a higher share of ncRNA misclassifications can be observed as compared to mRNA. This problem is more common in plant species in contrast to animal. Specifically, the majority of mRNA misclassifications are observed in CNIT *G. max*, CNIT *M. truncatula*, CNIT *C. elegans*, CPC2 *A. thaliana*, CPPred Integrated, LGC *M. musculus*, LGC *O. sativa*, PLncPRO *G. gallus*, PLncPRO *V. vinifera*, RNAC *A. thaliana* and RNAplonc *S. tubersum* datasets. In contrast, ncRNA misclassifications can be seen in CPAT *H. sapiens*, CPC2 *D. rerio*, CPPred *D. rerio*, FEElnc *D. melanogaster*, LGC *M. musculus*, LncFinder *G. gallus*, LncFinder *D. rerio*, PLncPRO *G. gallus*, PLncPRO *V. vinifera* and RNAplonc *S. tubersum* datasets

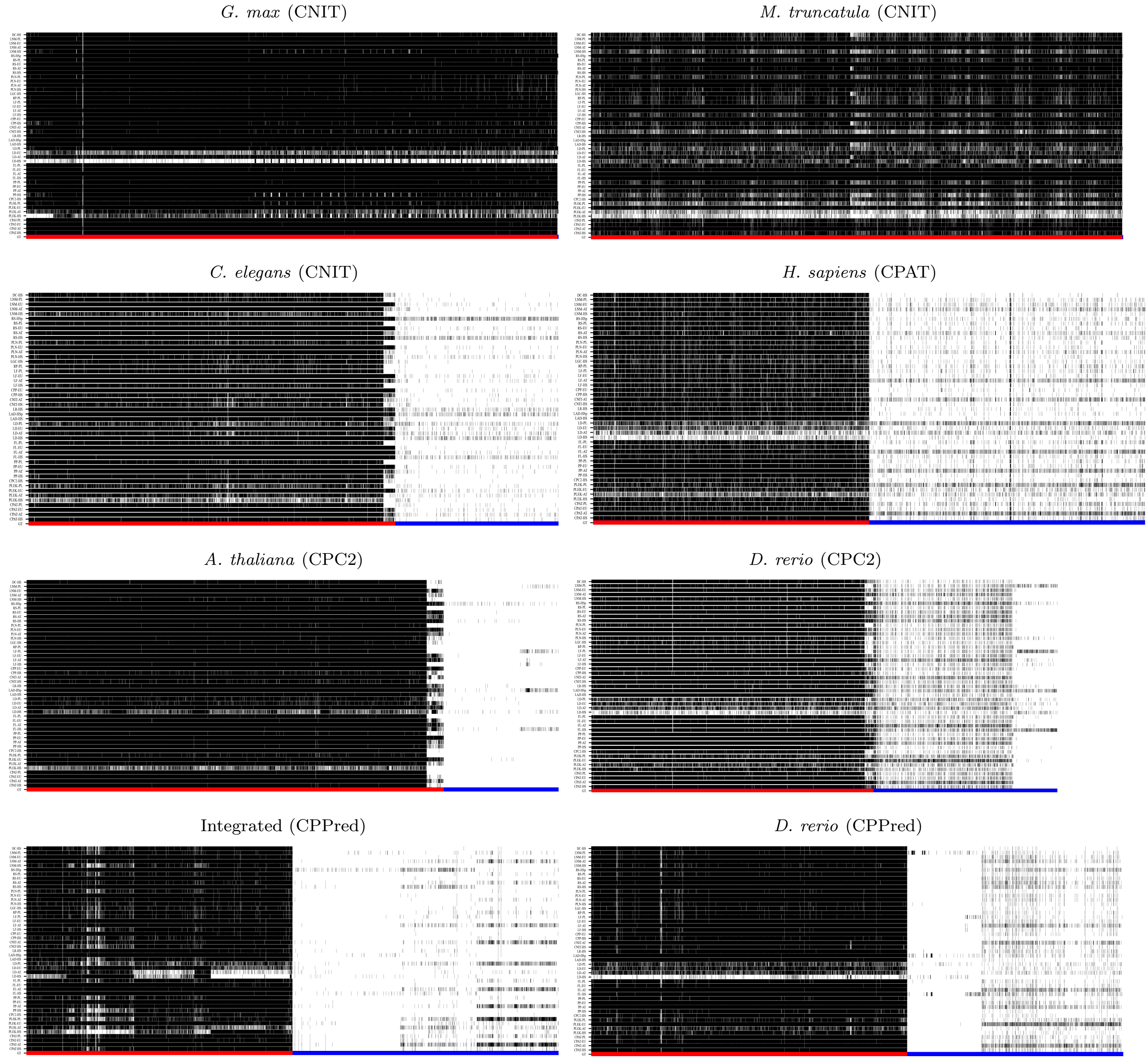

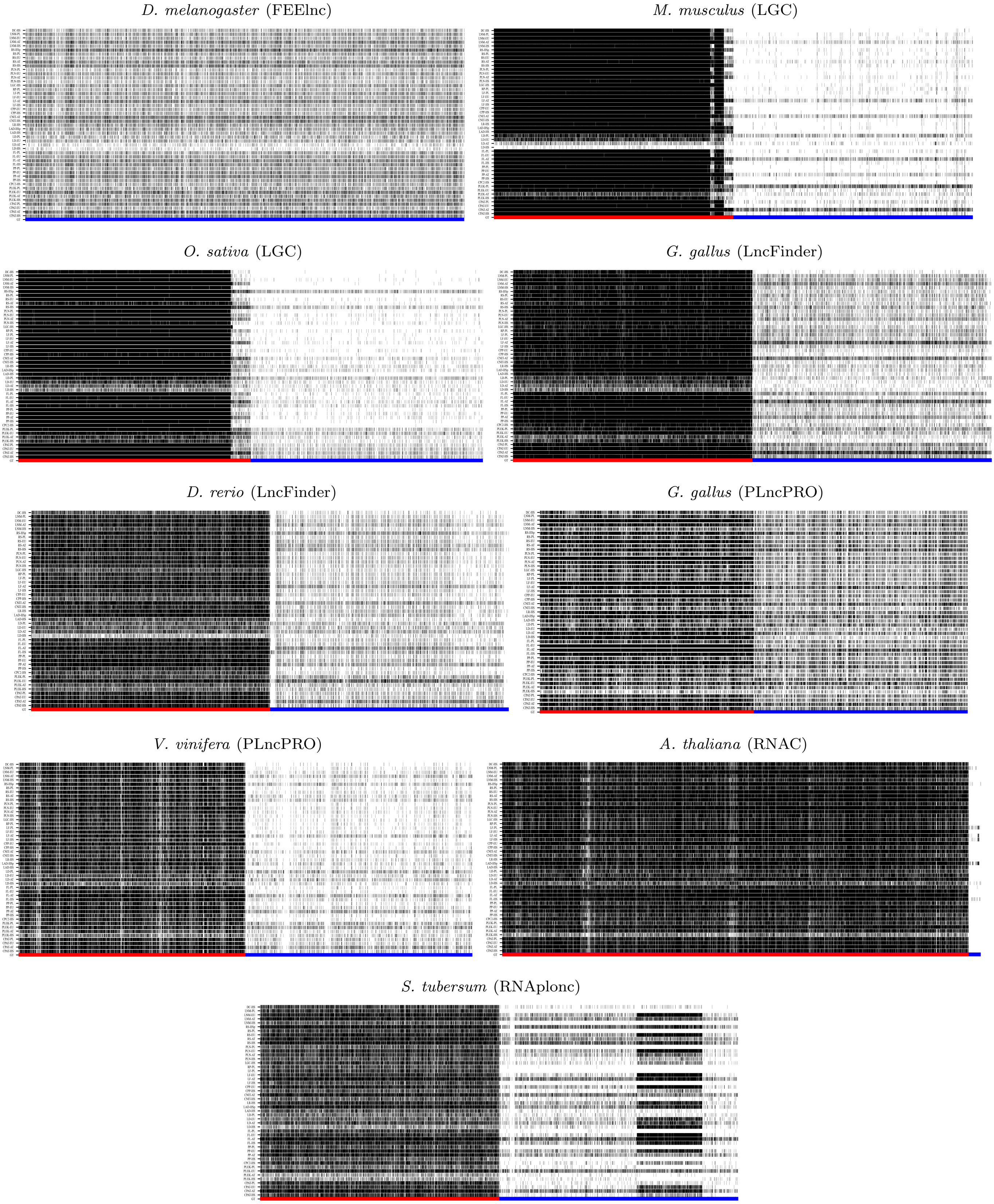

Figure S2: Classification decisions of models on 17 additional datasets. The red color represents the true mRNA, blue represents the true ncRNA, black represents the predicted mRNAs by all models and white represents the predicted ncRNAs by all models

4 Constructing a new challenging classification dataset

Table S1 presents the outcomes of six holdout tools on a sample of six datasets from the benchmark. These additional experiments are performed to validate the findings from the 18 tools. These outcomes from the 9 models corroborate with the key factors affecting the performance of current tools to predict the RNA types correctly.

Table S1: Performance of holdout tools on six samples datasets from the benchmark

Methods	Datasets	Accuracy	Precision	Recall	F1-score	MCC
	CPP-sIT	72.85	65.33	97.37	78.19	52.43
	LAD-MM	83.76	73.06	96.43	83.13	70.49
LI-HS	LR-HS	77.01	68.85	98.66	81.10	59.93
	RP-ST	80.77	82.23	78.49	80.32	61.59
	PP-lOS	70.88	100	70.88	82.96	0
	CPP-sIT	77.44	72.47	88.52	79.69	56.28
	LAD-MM	78.09	71.78	77.78	74.66	55.56
LI-AT	LR-HS	69.59	66.14	80.26	72.52	40.10
	RP-ST	67.31	83.95	42.80	56.70	39.71
	PP-lOS	57.82	100	57.82	73.27	0
	CPP-sIT	87.92	84.52	92.85	88.49	76.21
	LAD-MM	84.49	76.05	91.40	83.02	69.95
LI-EU	LR-HS	78.31	71.92	92.91	81.08	59.21
	RP-ST	65.20	81.28	39.49	53.15	35.43
	PP-lOS	53.89	100	53.89	70.03	0
	CPP-sIT	68.47	61.85	96.44	75.36	44.57
	LAD-MM	80.95	70.19	94.01	80.37	65.14
LI-PL	LR-HS	71.27	64.65	93.86	76.56	47.69
	RP-ST	86.04	80.72	94.70	87.16	73.19
	PP-lOS	67.56	100	67.56	80.64	0
	CPP-sIT	57.15	56.58	61.47	58.92	14.36
	LAD-MM	60.79	53.55	41.54	46.79	16.86
BI-HS	LR-HS	58.04	58.44	55.69	57.03	16.10
	RP-ST	62.87	65.21	55.18	59.78	26.05
	PP-lOS	28.18	100	28.18	43.97	0
	CPP-sIT	66.37	92.69	35.53	51.37	41.58
	LAD-MM	82.24	98.70	57.95	73.03	65.89
CRE-PL	LR-HS	70.31	98.30	41.34	58.21	49.85
	RP-ST	59.81	83.83	24.31	37.69	27.86
	PP-lOS	35.13	100	35.13	51.99	0
	CPP-sIT	84.94	87.69	81.30	84.37	70.07
	LAD-MM	91.97	92.56	87.70	90.07	83.42
PGF-HS	LR-HS	96.34	96.77	95.89	96.33	92.69
	RP-ST	66.13	83.41	40.27	54.32	37.70
	PP-lOS	46.37	100	46.37	63.36	0
	CPP-sIT	85.83	83.23	89.73	86.36	71.87
	LAD-MM	92.47	90.02	92.05	91.03	84.56
LMD-HS	LR-HS	92.54	88.60	97.66	92.91	85.53
	RP-ST	67.91	82.08	45.82	58.81	39.92
	PP-lOS	52.04	100	52.04	68.45	0
	CPP-sIT	48.15	48.08	46.43	47.24	-3.71
	LAD-MM	50.92	42.08	48.58	45.10	1.14
NCR-HS	LR-HS	51.41	51.44	50.51	50.97	2.83
	RP-ST	51.45	51.54	48.65	50.06	2.91
	PP-lOS	48.82	100	48.82	65.61	0

Figure S3 presents the distributions of sequence length as well as ORFs and their length in the RNAChallenge dataset. All sequences have been used to measure the distributions for the plot. To identify ORFs in transcripts, NCBI ORFfinder tool has been used with "ATG" start codon and minimum codon length of 30.

References

[1] Liguo Wang, Hyun Jung Park, Surendra Dasari, Shengqin Wang, Jean-Pierre Kocher, and Wei Li. Cpat: Coding-potential assessment tool using an alignment-free logistic regression model. *Nucleic acids research*, 41(6):e74–e74, 2013.

[2] Aimin Li, Junying Zhang, and Zhongyin Zhou. Plek: a tool for predicting long non-coding rnas and messenger rnas based on an improved k-mer scheme. *BMC bioinformatics*, 15(1):1–10, 2014.

[3] Siyu Han, Yanchun Liang, Ying Li, and Wei Du. Lncident: a tool for rapid identification of long noncoding rnas utilizing sequence intrinsic composition and open reading frame information. *International journal of genomics*, 2016, 2016.

[4] Yu-Jian Kang, De-Chang Yang, Lei Kong, Mei Hou, Yu-Qi Meng, Liping Wei, and Ge Gao. Cpc2: a fast and accurate coding potential calculator based on sequence intrinsic features. *Nucleic acids research*, 45(W1):W12–W16, 2017.

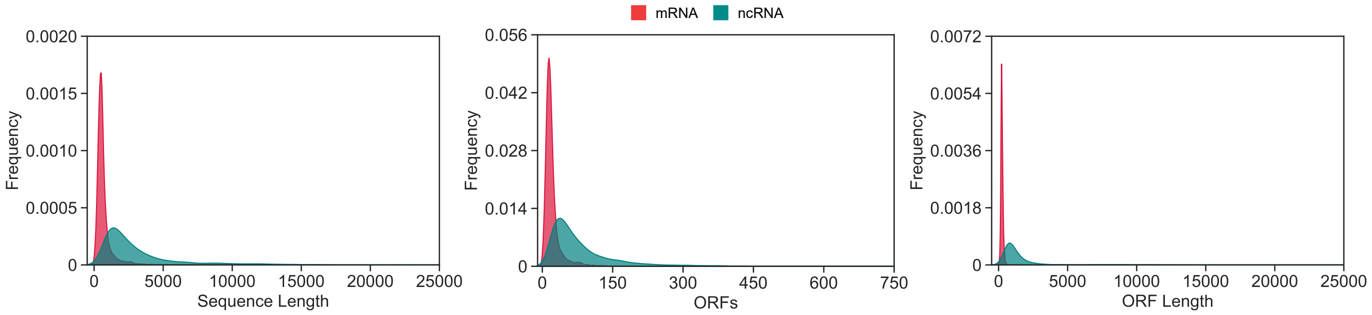

Figure S3: Distribution of sequence length as well as ORFs and their length in RNAChallenge dataset

[5] Lei Kong, Yong Zhang, Zhi-Qiang Ye, Xiao-Qiao Liu, Shu-Qi Zhao, Liping Wei, and Ge Gao. Cpc: assess the protein-coding potential of transcripts using sequence features and support vector machine. *Nucleic acids research*, 35(suppl_2):W345–W349, 2007.

[6] Urminder Singh, Niraj Khemka, Mohan Singh Rajkumar, Rohini Garg, and Mukesh Jain. Plncpro for prediction of long non-coding rnas (lncrnas) in plants and its application for discovery of abiotic stress-responsive lncrnas in rice and chickpea. *Nucleic acids research*, 45(22):e183–e183, 2017.

[7] Valentin Wucher, Fabrice Legeai, Benoit Hedan, Guillaume Rizk, Lætitia Lagoutte, Tosso Leeb, Vidhya Jagannathan, Edouard Cadieu, Audrey David, Hannes Lohi, et al. Feelnc: a tool for long non-coding rna annotation and its application to the dog transcriptome. *Nucleic acids research*, 45(8):e57–e57, 2017.

[8] Hugo W Schneider, Taina Raiol, Marcelo M Brigido, Maria Emilia MT Walter, and Peter F Stadler. A support vector machine based method to distinguish long non-coding rnas from protein coding transcripts. *BMC genomics*, 18(1):1–14, 2017.

[9] Cheng Yang, Longshu Yang, Man Zhou, Haoling Xie, Chengjiu Zhang, May D Wang, and Huaiqiu Zhu. Lncadeep: an ab initio lncrna identification and functional annotation tool based on deep learning. *Bioinformatics*, 34(22):3825–3834, 2018.

[10] Steven T Hill, Rachael Kuintzle, Amy Teegarden, Erich Merrill III, Padideh Danaee, and David A Hendrix. A deep recurrent neural network discovers complex biological rules to decipher rna protein-coding potential. *Nucleic acids research*, 46(16):8105–8113, 2018.

[11] Junghwan Baek, Byunghan Lee, Sunyoung Kwon, and Sungroh Yoon. Lncrnanet: long non-coding rna identification using deep learning. *Bioinformatics*, 34(22):3889–3897, 2018.

[12] Eric Augusto Ito, Isaque Katahira, Fábio Fernandes da Rocha Vicente, Luiz Filipe Protasio Pereira, and Fabrício Martins Lopes. Basinet—biological sequences network: a case study on coding and non-coding rnas identification. *Nucleic acids research*, 46(16): e96–e96, 2018.

[13] Caitlin MA Simopoulos, Elizabeth A Weretilnyk, and G Brian Golding. Prediction of plant lncrna by ensemble machine learning classifiers. *BMC genomics*, 19(1):1–11, 2018.

[14] Jin-Cheng Guo, Shuang-Sang Fang, Yang Wu, Jian-Hua Zhang, Yang Chen, Jing Liu, Bo Wu, Jia-Rui Wu, En-Min Li, Li-Yan Xu, et al. Cnit: a fast and accurate web tool for identifying protein-coding and long non-coding transcripts based on intrinsic sequence composition. *Nucleic acids research*, 47(W1):W516–W522, 2019.

[15] Xiaoxue Tong and Shiyong Liu. Cppred: coding potential prediction based on the global description of rna sequence. *Nucleic acids research*, 47(8):e43–e43, 2019.

[16] Siyu Han, Yanchun Liang, Qin Ma, Yangyi Xu, Yu Zhang, Wei Du, Cankun Wang, and Ying Li. Lncfinder: an integrated platform for long non-coding rna identification utilizing sequence intrinsic composition, structural information and physicochemical property. *Briefings in bioinformatics*, 20(6):2009–2027, 2019.

[17] Tatianne da Costa Negri, Wonder Alexandre Luz Alves, Pedro Henrique Bugatti, Priscila Tiemi Maeda Saito, Douglas Silva Domingues, and Alexandre Rossi Paschoal. Pattern recognition analysis on long noncoding rnas: a tool for prediction in plants. *Briefings in bioinformatics*, 20(2):682–689, 2019.

[18] You Duan, Wanting Zhang, Yingyin Cheng, Mijuan Shi, and Xiao-Qin Xia. A systematic evaluation of bioinformatics tools for identification of long noncoding rnas. *RNA*, 27(1):80–98, 2021.

[19] Guangyu Wang, Hongyan Yin, Boyang Li, Chunlei Yu, Fan Wang, Xingjian Xu, Jiabao Cao, Yiming Bao, Liguo Wang, Amir A Abbasi, et al. Characterization and identification of long non-coding rnas based on feature relationship. *Bioinformatics*, 35(17): 2949–2956, 2019.

[20] Shuai Liu, Xiaohan Zhao, Guangyan Zhang, Weiyang Li, Feng Liu, Shichao Liu, and Wen Zhang. Predlnc-gfstack: a global sequence feature based on a stacked ensemble learning method for predicting lncrnas from transcripts. *Genes*, 10(9):672, 2019.

[21] Lei Cao, Yupeng Wang, Changwei Bi, Qiaolin Ye, Tongming Yin, and Ning Ye. Prelnc: An accurate tool for predicting lncrnas based on multiple features. *Genes*, 11(9):981, 2020.

[22] Antonio P Camargo, Vsevolod Sourkov, Gonçalo A G Pereira, and Marcelo F Carazzolle. Rnasamba: neural network-based assessment of the protein-coding potential of rna sequences. *NAR genomics and bioinformatics*, 2(1):lqz024, 2020.

[23] Xiao-Nan Fan, Shao-Wu Zhang, Song-Yao Zhang, and Jin-Jie Ni. lncrna_mdeep: an alignment-free predictor for distinguishing long non-coding rnas from protein-coding transcripts by multimodal deep learning. *International journal of molecular sciences*, 21(15): 5222, 2020.

[24] Sen Yang, Yan Wang, Shuangquan Zhang, Xuemei Hu, Qin Ma, and Yuan Tian. Ncresnet: noncoding ribonucleic acid prediction based on a deep resident network of ribonucleic acid sequences. *Frontiers in genetics*, 11:90, 2020.

[25] H Busra Cagirici, S Galvez, Taner Z Sen, and Hikmet Budak. Lncmachine: a machine learning algorithm for long noncoding rna annotation in plants. *Functional & Integrative Genomics*, 21(2):195–204, 2021.

[26] Yu Zhang, Cangzhi Jia, Melissa Jane Fullwood, and Chee Keong Kwoh. Deepcpp: a deep neural network based on nucleotide bias information and minimum distribution similarity feature selection for rna coding potential prediction. *Briefings in bioinformatics*, 22 (2):2073–2084, 2021.
